# The Role of Natural Products in Immunopharmacology

**DOI:** 10.3390/ijms25179256

**Published:** 2024-08-27

**Authors:** Giulia Accardi, Danila Di Majo, Anna Aiello

**Affiliations:** 1 Section of General Pathology, Department of Biomedicine, Neuroscience and Advanced Diagnostics, School of Medicine, University of Palermo, 90134 Palermo, Italy; anna.aiello@unipa.it; 2 Section of Human Physiology, Department of Biomedicine, Neuroscience and Advanced Diagnostics, School of Medicine, University of Palermo, 90127 Palermo, Italy; danila.dimajo@unipa.it

The Special Issue “The Role of Natural Products in Immunopharmacology”, edited by Giulia Accardi, Danila Di Majo, and Anna Aiello focuses on the crucial role of natural products and their related components in treating various disorders, emphasizing their applications in the pharmacological and nutraceutical fields. 

Historically, natural products have been used to treat a wide range of conditions. However, scientific validation of these traditional uses is often lacking. Bridging this gap is essential for the development of new pharmaceuticals or functional foods. 

In this Special Issue, we have collected articles that describe natural products isolated using molecular approaches, with applications aimed at understanding the mechanisms of action in improving different kinds of disorders, including immune pathologies. The focus has been on natural compounds and their derivatives, rather than mere extracts, to provide a more precise understanding of their biological effects.

The paper edited by Scicchitano and colleagues investigated the dual role of oleuropein, a compound found in olive leaves, in ovarian and breast cancer cells [1]. Their study demonstrated that high doses of oleuropein exhibit anti-proliferative and pro-apoptotic effects on cancer cells. Conversely, low doses were found to reduce oxidative stress and maintain cell viability without inducing cell death. This research highlights oleuropein’s potential as both an antioxidant and a pro-oxidant, depending on the dosage, and reinforces the evidence towards the anti-cancer properties of oleuropein, as well as, its synergic effect with chemotherapeutic drugs such as doxorubicin and cisplatin,, providing new insights into its application in cancer therapy [2,3]. However, despite the promising preclinical results, further investigations in the clinical field are required to confirm its role as a chemotherapeutic agent, also considering the variability of its bioavailability and metabolism, which represents a challenge for further oleuropein research.

Jędrzejewski and co-authors reviewed the therapeutic potential of *Coriolus versicolor* (CV), a mushroom used in traditional Chinese medicine [4]. They focused on the immunomodulatory effects of CV’s polysaccharopeptides, such as PSP and PSK, in cancer and viral infections, including COVID-19. The study underscores the mushroom’s ability to enhance immune responses and provides a comprehensive overview of its mechanisms in anti-cancer and anti-viral contexts. Before considering a therapeutic application of CV in humans, it is crucial to conduct other research on mushroom derivatives compounds, especially regarding potential adverse effects. Understanding the possible interactions and side effects is essential for safe and effective use. For example, recent studies indicating adverse effects in a population undergoing chemotherapy or radiotherapy for colon–rectal cancer underscore the need for caution [5].

Pojero et al., exploring the world of the phytochemicals extracted from the olive tree, examined the anti-inflammatory properties of oleuropein, and its derivative hydroxytyrosol, particularly in the context of inflammaging [6]. The review highlights the potential of these compounds to modulate tissue inflammation and oxidative stress, proposing them as effective nutraceutical interventions to mitigate age-related inflammatory conditions. Despite the considerable potential of hydroxytyrosol for human health, its use in clinical practice is still not completely safe due to the lack of standardized dosages, and there are no long-term studies on its use at high doses. Regarding its bioavailability, its absorption is variable, conditioned by genetic, dietary and metabolic factors. Additionally, due to its rapid metabolism, it requires frequent administration at high dosages or in specific formulations that improve its bioavailability [7,8]. Unfortunately, the clinical trials conducted to investigate the effect of oleuropein in humans are limited and involve only a small number of subjects [9]. Finally, its purity in the formulations administered should also be considered.

Roszczyk and colleagues reviewed the health benefits of polysaccharides derived from the shiitake mushroom (*Lentinula edodes*). These compounds exhibit significant anti-cancer, antioxidant, antimicrobial, and immunomodulatory properties [10]. The article discusses how various extraction methods affect the biological activity of these polysaccharides and highlights their potential applications in enhancing immune responses in both animal models and humans. While generally considered safe for consumption, some authors have reported adverse effects such as shiitake dermatitis, a skin reaction caused by consuming raw or undercooked shiitake mushrooms. Additionally, some individuals may experience gastrointestinal discomfort. More comprehensive studies and clinical trials are needed to fully understand the safety profile and potential side effects of long-term consumption of *Lentinula edodes*, especially in therapeutic doses.

This project collectively underscores the immense potential of specific natural products in the treatment and management of various health conditions, particularly immune disorders and age-related inflammatory diseases, as previously deeply investigated [11,12,13,14]. By providing scientific evidence on the mechanisms of action of these natural compounds, the research highlights their promise in pharmaceutical and nutraceutical developments and the need for further research in the field of natural product-based therapeutics. When evaluating the use of these products in the pharmaceutical and nutritional fields, it is essential to consider the interference that polyphenolic compounds and, more generally, natural products, may have with a variety of drugs. Therefore, large-scale clinical studies and in vitro and in vivo studies are needed to confirm their therapeutic properties. Additional research is also necessary to assess their ability to interact with the mechanisms of action of other drugs, particularly their effect on cytochrome P450.
